# Active oscillations in microscale navigation

**DOI:** 10.1007/s10071-023-01819-5

**Published:** 2023-09-04

**Authors:** Kirsty Y. Wan

**Affiliations:** 1 Living Systems Institute, University of Exeter, Stocker Road, Exeter, EX4 4QD UK; 2 Department of Mathematics and Statistics, University of Exeter, Stocker Road, Exeter, EX4 4QL UK

**Keywords:** Navigation, Motility, Biological oscillations, Basal cognition, Evolution, Behaviour

## Abstract

Living organisms routinely navigate their surroundings in search of better conditions, more food, or to avoid predators. Typically, animals do so by integrating sensory cues from the environment with their locomotor apparatuses. For single cells or small organisms that possess motility, fundamental physical constraints imposed by their small size have led to alternative navigation strategies that are specific to the microscopic world. Intriguingly, underlying these myriad exploratory behaviours or sensory functions is the onset of periodic activity at multiple scales, such as the undulations of cilia and flagella, the vibrations of hair cells, or the oscillatory shape modes of migrating neutrophils. Here, I explore oscillatory dynamics in basal microeukaryotes and hypothesize that these active oscillations play a critical role in enhancing the fidelity of adaptive sensorimotor integration.

## Introduction

The ability to move in a seemingly purposeful, goal-oriented way even in the presence of dynamic environmental perturbations is sometimes assumed to be a sign of cognition or even intelligence. Indeed, the most ancestral purpose of the nervous system of living organisms may have been to control self-motion (i.e., motility) (Wan and Jékely 2021; Jékely et al. 2015; De Wiljes et al. 2015; Keijzer et al. 2013). Pioneering studies in diverse animal species have demonstrated how multiple sensory cues, vision, olfaction, audition, magnetoreception, etc. are integrated actively by the organism’s sensorimotor system to regulate behaviour and improve navigational performance (Vickers 2000; Collett and Graham 2004; Geva-Sagiv et al. 2015). Typically, distinct modalities of sensory input are encoded in different regions of the brain (and by different types of neurons) that together provide the animal with essential information about its location within the environment, the layout, or ‘map’ of its surroundings—including any potential obstacles, its speed of movement, trajectory heading, and the precise orientation of its own ‘self’ in three-dimensional space (Collett and Graham 2004; Frost and Mouritsen 2006; Toledo et al. 2020).

Compare and contrast this to the microscopic realm, with no paucity of examples of goal-oriented navigation strategies found in diverse microbial species. That microorganisms, including single cells, also have the capacity to receive, to process, and to perceive information, potentially also integrating multiple distinct sensory cues, is becoming increasingly evident (Lyon 2015). These traits were openly recognised by influential early researchers (Jennings 1902; Binet 1894), but it is only with the advent of recent bioimaging technologies (high-speed cameras, microscopes with high resolving power, etc.), that these processes and hypotheses can be queried and critiqued in detail.

The act of navigation (of an organism or animal) should be distinguished from simple migration from point A to point B, for the former includes active sampling of the environment, and successful negotiation of unpredictable or unfamiliar terrains. In both air and water, behaving animals routinely exploit and adapt to complex chemical and fluid dynamic landscapes in response to a (possibly patchy or transient) nutrient or odour source. Sampling is generally not a random process but in some cases has been shown to obey a closed-loop motor programme. Oscillations of hair-like appendages such as whiskers and antennae function to replenish the local air or water to increase olfactory function (Claverie et al. 2022). Oscillations of motile cilia in the zebrafish nose have been shown to produce flows that improve the temporal resolution of olfactory sensing (Reiten et al. 2017). Steering or directional reorientations can emerge as a consequence of the feedback between oscillations that alter the amount of exposure to the environmental stimulus, and the stimulus that can in turn perturb the dynamics of the oscillator. Such a mechanism has been demonstrated in the *Drosophila* larval crawling behaviour, which exhibits lateral oscillations involving significant left-to-right sweeping movements of its head (containing the chemosensory organ of the larva) (Wystrach et al. 2016; Gomez-Marin et al. 2011). In two phylogenetically distant species of navigating ants, it was shown that they exhibit movement patterns with a persistent, underlying oscillatory component that is at least tenfold slower than that of the ants’ natural stride frequency (Clement et al. 2023). In mammals, the so-called theta oscillations that appear in electrical recordings of brain activity have been shown to play a key role in spatial navigation and memory, perhaps to promote the synchronization of neuronal firing (Bender et al. 2015).

Beyond examples relevant for navigation and sensing, it is clear that inherently oscillatory phenomena are highly widespread in biological systems as a signature of a complex dynamical system (Kruse and Jülicher 2005). Spontaneous waves and oscillations may simply reflect the underlying dynamic organisation or boundary conditions of the system, and do not have a purpose. For instance, spontaneous noisy oscillations have been observed in minimal reconstituted acto-myosin networks under elastic loading, comprising a single actin filament and only tens of myosin motors (Plaçais et al. 2009). In other cases, oscillations do have a clear functional role. In the clock-and-wavefront model, molecular segmentation clocks—oscillating patterns of gene expression—in the pre-somitic mesoderm underlie vertebrate somitogenesis, leading to cellular differentiation (e.g., into endothelium, dermis, skeletal muscle, tendon, and cartilage) (Oates et al. 2012; Chou et al. 2022). In the syncytial protist *Physarum polycephalum*, famed for its ability to solve mazes and respond to chemical cues, cellular migration is tightly coupled to the vascular network of the organism, resulting in pronounced contraction–relaxation cycles and oscillatory patterns of fluid flow. These flows are conjectured to provide an alternative route to memory and information storage that is distinct from the evolution of nervous systems (Boussard et al. 2021; Alim et al. 2017; Reid 2023).

The purpose of this essay is to explore and clarify the putative functional role of active oscillations in the context of microscale navigation strategies, particularly in aquatic environments. I will discuss the necessity and inevitability of oscillatory behaviours as a means to generate locomotion in an inertia-less regime (relevant for very small organisms), and the physical consequences of these actions, particularly for sensing. I hope that this motivates new insights into how organisms, both macroscopic and microscopic, make important decisions about *where* to go and *how* to get there, and how these capabilities originated in the first instance in the microscopic world.

## Oscillatory dynamics at the microscopic scale

### Oscillations as a means of moving

**Fig. 1:**
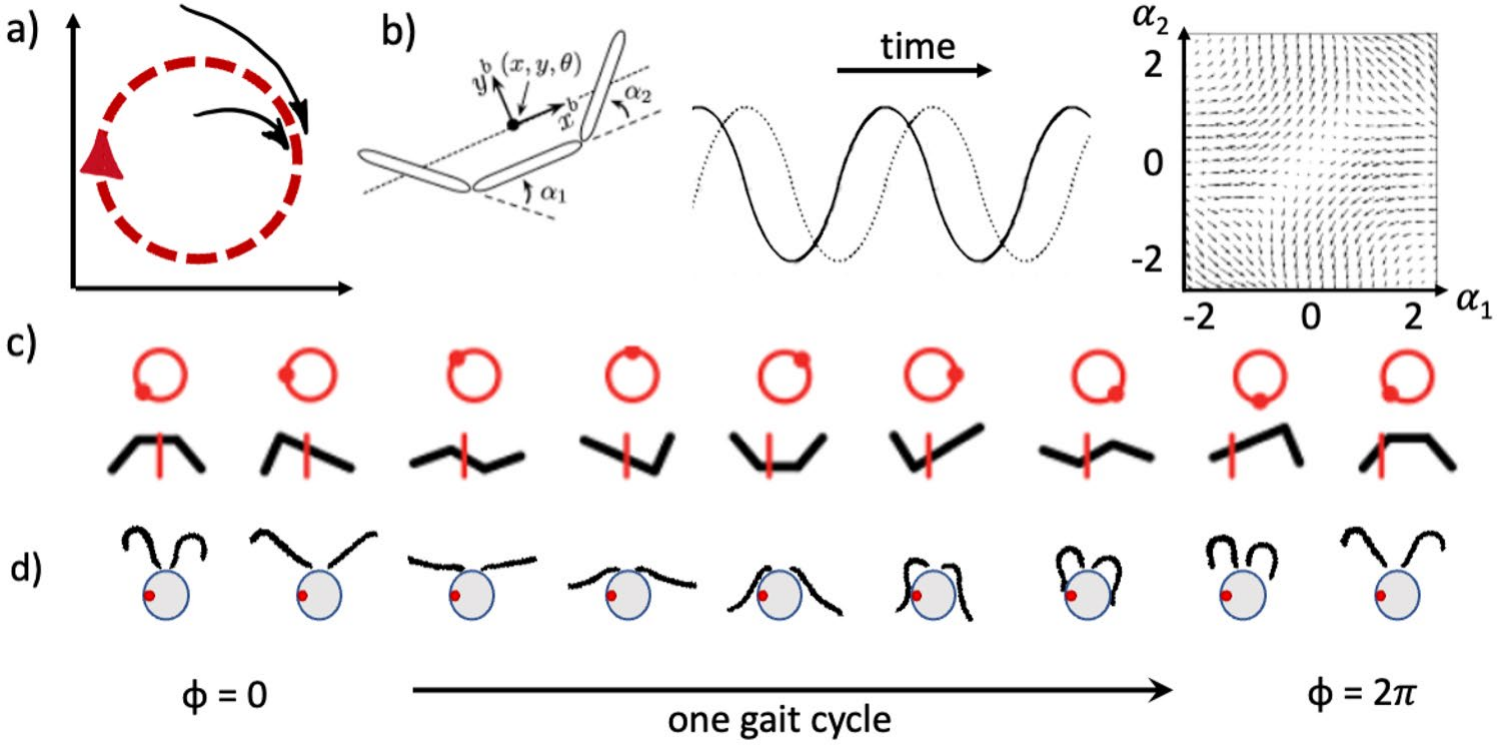
Conceptual summary of geometric mechanics in action at the microscale. **a** Limit cycle dynamics (red arrow) underlie diverse motility mechanisms; these are often stable to perturbations (black arrows). **b** Oscillations are natural due to the need to create a periodic gait. The displacement maps linearly to the shape velocity via a local connection matrix (here plotted for forward motion), where shape is represented by a 2D shape-space of angles $\alpha =(\alpha _1,\alpha _2$). The distance travelled is then proportional to the contour integral along the gait in shape-space, and hence the frequency of gait actuation. [Figure adapted from Hatton et al. (2013), Gong et al. (2016), and courtesy of Daniel Goldman.] A basic artificial three-link swimmer generates a non-reciprocal sequence of shapes that can achieve net swimming (**c**) [red vertical reference line shows net rightward displacement), while the biflagellate alga *Chlamydomonas* actuates its two cilia in a coordinated breaststroke (**d**) (data obtained by the author) (color figure online)

From the flapping tails of fish, to the rhythmic writhing of eels and snakes, diverse animal species achieve locomotion by stereotyped and periodic actuation of body parts. When the wavelength of the gait is shorter than the body length, the swimmer is generally known as an oscillatory swimmer, if longer, it may instead be classified as an undulatory swimmer (Smits 2019). Cyclic behaviours are natural or indeed inevitable, since they are needed to return the body part or appendage to its initial position, so that the next step, wave, or gait cycle can begin anew (Purcell 1977; Gallistel et al. 1983) (Fig. 1).

At the microscopic scale, organisms rely mainly on viscous drag, rather than inertia, for self-propulsion. For macroscopic animals, this is the difference between swimming in honey, rather than in water. Here, geometry dictates motion, and the gait is kinematic. It is not the rate of shape change, or how fast an appendage deforms, but the extent of deformation that is most important (Hatton et al. 2013). Simply put, the swimmer’s speed is linearly proportional to its shape velocity (Shapere and Wilczek 1989). For example, consider a three-link swimmer, first hypothesized by Purcell (1977), whose time-dependent shape is completely characterised by a vector of joint angles ${\varvec{\alpha }}=(\alpha _1,\alpha _2)$. The swimmer’s shape velocity would then be given by $\textrm{d}{\varvec{\alpha }}/\textrm{d}t$. A $3\times 2$ matrix, called the *connection*, then relates the swimmer’s speed in $(x,y,\theta )$-space with ${\varvec{\alpha }}$ (Fig. 1b). The swimming velocity is maximised when the shape velocity and the connection vectors are parallel. In a properly parameterised shape space, such as $(\alpha _1,\alpha _2)$, a gait (i.e., sequence of cyclic shape changes), produces closed curves that enclose a finite area, leading to propulsion. Net swimming is a consequence of many small (infinitesimal) translations and rotations generated by the organism’s shape changes, summed over time. For the Purcell swimmer, an undulatory gait sequence where the two angles are 1/4-cycle phase-lagged will trace out a circle in shape-space (Fig. 1c), to produce net displacement in the positive-x direction (Hatton et al. 2013; Wiezel and Or 2016). Such a planar 3-link swimmer has been shown to be controllable in 2D in a control theoretic sense, meaning that every final state is reachable from some initial state, and that the optimal strategy for swimming is indeed to to execute periodic sequences of strokes (Giraldi et al. 2013). More generally, symmetry is broken—and a transition to a more ordered but asymmetrical state is achieved—in the deformation sequence, either by propagating a wave, which is inherently directional, or by generating an asymmetric beat pattern (Fig. 1d). This is a consequence of the lack of time-dependence in the Stokes’ equations, which govern motion in a viscosity-dominated regime. Playing the same stroke pattern backwards in time solves the same equations and leads to the same motion, but in reverse. This is known as *Stokes’ reversibility*, which microswimmers must overcome to achieve net propulsion.

**Fig. 2:**
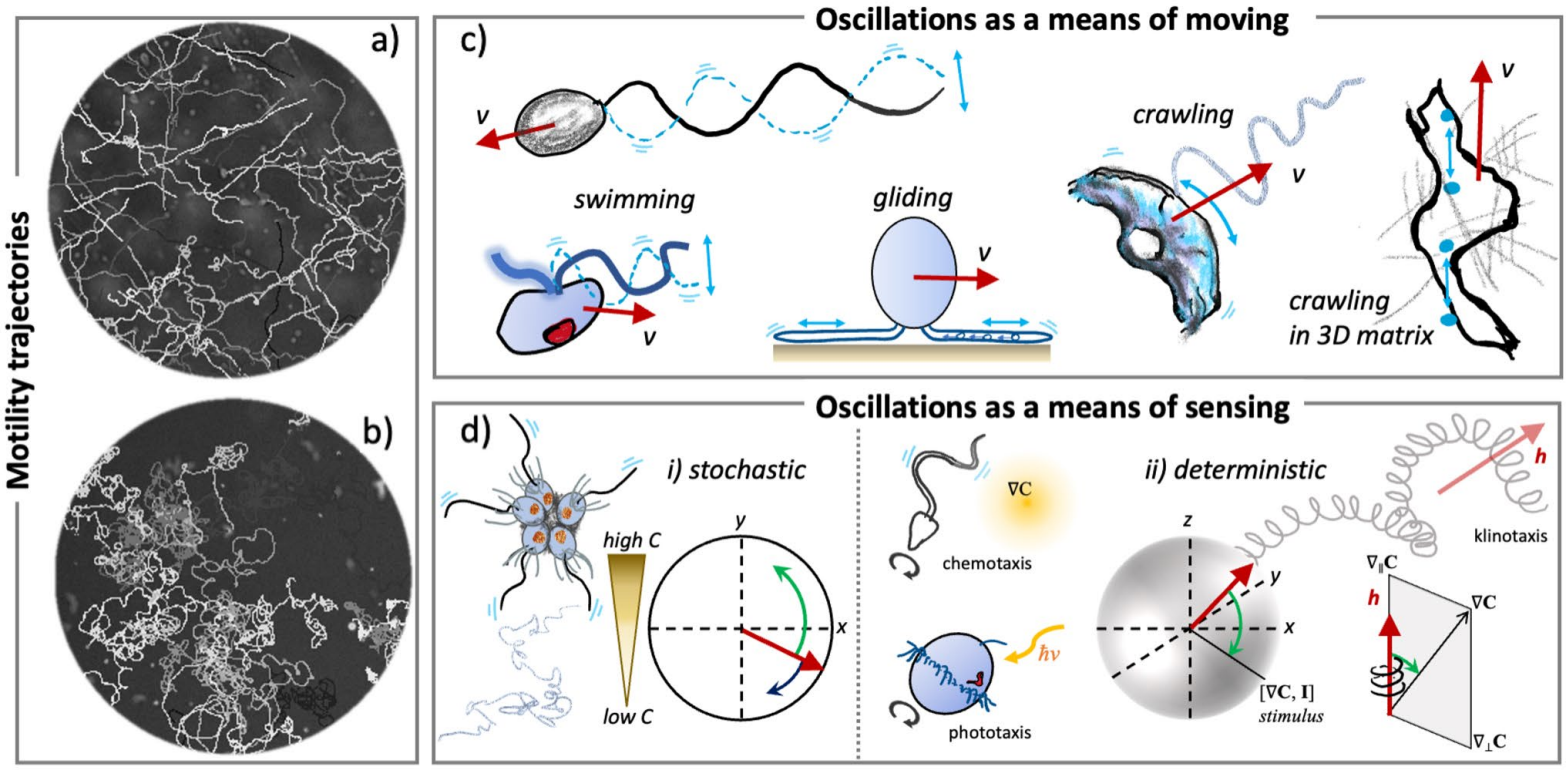
Oscillations in microscale navigation are ubiquitous, but do they serve a purpose? **a**, **b** Motility trajectories of two different strains of *Chlamydomonas reinhardtii*, a model biflagellate alga, observed under the microscope. **c** Examples of motility strategies that rely on the active oscillations of body parts: swimming using cilia and flagella, gliding on substrates, bipedal crawling of keratocytes, and eukaryotic cell migrating as a pair of oscillating force dipoles. (Red arrows indicate direction of migration.) **d** Examples of sensing strategies that rely on the active oscillations of cilia: stochastic aerotaxis of choanoflagellates (the colony makes as many correct as incorrect turns), deterministic (helical) klinotaxis in many small eukaryotes, and sperm cells (the superhelical paths of the organism tend to align with the stimulus direction) (color figure online)

This basic principle is exploited by various microorganisms (as well as bioinspired artificial or robotic swimmers) that can actuate different appendage types (flagella, archaella, or cilia) (Bondoc-Naumovitz et al. 2023; Diaz et al. 2021; Lim et al. 2022). Whereas bacterial flagella and archaella (the flagella of archaeal species) are rigid helices that can only be rotated from one end, cilia are much larger, structurally more complex, and only found in eukaryotes where they display more degrees of mechanical freedom (Wan and Jékely 2021; Beeby et al. 2020). Although these appendage types differ fundamentally in their mode of operation and actuation mechanism, they have in common a slender shape that experiences anisotropic drag as segmental elements are moved through a fluid. In diverse organisms, appendages are arranged in various configurations, and actuated in a periodic manner through fluids and other complex friction-dominated environments (Fig. 2a, b).

Even motile cell types that do not have permanent appendages employ temporary actin-based protrusions called pseudopodia which grow and retract around the cell, to produce the symmetry breaking and body deformations that are necessary for net motility. As fish keratocytes crawl on substrates, the coupling between elasticity and adhesion gives rise to distinctive oscillations in shape akin to a ‘bipedal’ gait (Barnhart et al. 2010). Other eukaryotic cells that routinely navigate through tubes and confined spaces exhibit increased directional persistence in tight confinement than when unconstrained. In an endothelial cell line, physical confinement increased the levels of intracellular Ca$^{2+}$, which regulates an underlying molecular clock (with negative feedback) that produces oscillations in RhoA GTPase activity and alters cell migration speed (Lee et al. 2022). Coherent cell-scale actin flows can help polarise animal cells for directional movement (Reversat et al. 2020), but may not be the dominant mechanism for cells migrating in physiological 3D matrices. In such matrix networks, migrating cells exhibited cyclic deformations and speed oscillations that can be captured by a model of the cell as a pair of oscillating force dipoles (one on either side of the nucleus) that breaks symmetry via a phase shift in time (Godeau et al. 2022) (Fig. 2c). In these types of dynamics, the net migration speed over one cycle is proportional to the gait frequency.

More unusual migration strategies are found in diverse protists. The process by which euglenid species produce extreme but periodic deformations of their entire bodies is known as metaboly, where flexible strips of pellicle, a flexible proteinaceous layer covering the outer cell membrane, are sheared helically past each other (Arroyo et al. 2012). This produces a relatively slow (only 1–2 $\upmu$m/s) swimming speed, compared to what is typical of cilia-driven motility (often on the order of 100 $\upmu$m/s). In Plasmodium, single-celled parasites of the phylum Apicomplexa, sporozoites exhibit gliding motility on substrates, producing striking circular trajectories on glass slides, but helical paths when moving in 3D. The physical mechanism for this is unclear but is thought to be related to the basic chiral shape of the cell (Yahata et al. 2021). The periodicity of the trajectories appears to be strongly correlated with the oscillations in intracellular Ca$^{2+}$ levels (Carey et al. 2014). The model biflagellate alga *Chlamydomonas* also glides on surfaces. This form of motility repurposes an ancient mechanism known as Intraflagellar Transport (IFT) by which proteins are transported back and forth along the axoneme by molecular motors (kinesins and dyneins) (Bloodgood 2010). This mechanism does not rely on the propagation of bends along the axoneme, but instead membrane glycoproteins carried by the IFT machinery transiently adhere to the substrate. The motors responsible for cargo transport then exert pulling forces on the paused glycoproteins, resulting in gliding along the direction of the flagellum (Shih et al. 2013). Whole cells glide with both flagella fully outstretched (Fig. 2c), but how can symmetry be broken? It turns out that Ca$^{2+}$ signalling is compartmentalised and coordinated between the two flagella. In the trailing flagellum, elevated Ca$^{2+}$ promotes the disengagement of IFT particles from the flagella to enable the leading flagellum to win the tug of war and facilitate smooth gliding. On adherent surfaces, repetitive Ca$^{2+}$ elevations prevent the accumulation of paused IFT particles, thereby indirectly regulates the adhesion force (Bloodgood 2010; Fort et al. 2021).

### Oscillations as a means of sensing

In tracing the deep evolutionary origins of microorganisms, the ancestral cilium undoubtedly played an important role in sensing (Quarmby and Leroux 2010; Jékely 2009), though it is unclear which aspect of cilium function arose first: motility or sensing. Though motile cilia have become specialised for propelling flows, they are nevertheless adorned with ion channels and diverse capabilities to transduce and sense environmental signals. In the absence of external cues, the orderly beat of some motile cilia can be highly robust to biochemical noise due to background fluctuations, maintaining a sharply peaked frequency distribution in such species. In *Chlamydomonas*, changes in the cilia beat frequency spectrum are a direct readout of the cell’s physiological state, with slow oscillations in the instantaneous beat frequency occurring on a much slower timescale than flagellar beating itself, possibly due to fluctuations in intracellular Ca$^{2+}$ (Wan and Goldstein 2014). When the cilium is perturbed mechanically, its preferred or baseline beat dynamics can be recovered quickly, within characteristic time, and in a load-dependent manner (Wan and Goldstein 2014; Klindt et al. 2016).

Some motile cilia display repetitive movements that are distinct from normal ciliary beating in that they lack large-amplitude bending, instead undergoing periodic, small-amplitude vibrations. This vibrational mode has been reported in green algae (Wan and Goldstein 2018) and in the hyperoscillations of sperm flagella (Brokaw 2009; Kamimura and Kamiya 1989). Conversely, while most sensory (sometimes known as primary) cilia have lost their ability to beat, some specialised cilia types found in the vertebrate node (Yuan et al. 2015) and in the mechanosensory organs of marine invertebrates also vibrate at defined frequencies (Bezares-Calderón et al. 2020). Cilia vibrations are associated with the acute mechanosensory capabilities of marine algae (Wan and Goldstein 2018) and in the unusual auditory system of tree crickets (Mhatre 2015). Similarly, faint sounds are known to be actively amplified by mechanosensory hair bundles in the mammalian inner ear, by exploiting an oscillatory instability (where the physical system suddenly exhibits oscillations as a certain critical threshold in forcing is reached, see also further discussions in the section “Oscillations at system criticality”) (Camalet et al. 2000). The existence of an underlying frequency signature seems to be a universal feature in the above examples, and suggests that oscillations could contribute to sensory function.

One possibility lies in the ability of certain cells *to generate their own signals*, especially in cases where the external signal is weak (e.g., shallow chemical gradients) or cell size is small (Wan and Jékely 2021; Dusenbery 1998). Since oscillatory inputs (in contrast to steady-state signals) have high-information content, cells can overcome sensory limitations by generating self-oscillations and then convolving the self-originated signals with environmental information. According to an emerging perspective, crawling eukaryotic cells (e.g., immune cells) exploit self-generated gradients for steering and chemotaxis (Insall et al. 2022; Tweedy et al. 2020). Indeed, the same cell can both produce and respond to the signalling molecule or chemical, such as during autocrine signalling. When this occurs with a temporal feedback or relay (Insall et al. 2022), complex dynamics can emerge including coordinated behaviour across a population of cells.

Regardless of whether or not the motile cells have temporary protrusions or permanent appendages, they often follow saltatory trajectories reminiscent of the lateral oscillations of the behaving ants I described above. These dynamics may be further coupled to various types of taxes (Ehrengruber et al. 1996). In its single-cell amoeba stage, *Dictyostelium* undergoes repetitive shape changes as it crawls, which coincides with force-dependent changes in adhesion, to produce a stepping action. Even in the absence of external cues, this stepping largely conforms to a left–right–left alternating pattern of pseudopod extension with a period of 1–2 min (Bosgraaf and Van Haastert 2009). This non-random pattern of always turning away from the last turn results in a diffusive zigzag search pattern with long directional persistence, which may optimise sampling efficiency in unseen environments (Yang et al. 2011; Li et al. 2008). In biophysical models, zigzag trajectories where a particle hops with equal probability left or right but with some fixed or preferred turning angle produce large spatial diffusion constants and enhanced area of coverage (Schimansky-Geier et al. 2005). This search strategy has also been hypothesized to confer an evolutionary advantage in some organisms, such as in the case of the optimal foraging behaviour of the plankton *Daphnia* (Garcia et al. 2007).

Taken together, we may hypothesize that single cells (indeed sub-cellular structures) harness self-initiated oscillatory activity to create sensory reafference by generating significant relative motion between the self and the environment, and thereby achieve heightened sensing. These active sensing modalities may parallel well-established processes in the macroscopic world, as found in many marine invertebrates (Jékely et al. 2021) and in weakly electric fish (Hofmann et al. 2013). I will return to this point in the next section in the specific case of freely swimming microorganisms with helical motility patterns (Fig. 2d).

### Oscillations at system criticality

From a physical perspective, living systems are often said to be poised at criticality (Mora and Bialek 2011; Kinouchi and Copelli 2006), treading that metaphorical fine line between order and disorder. This precarious yet useful state of existence ensures that such systems are simultaneously robust to noise, and yet also able to respond to environmental changes in a timely manner. Excessive order can make a dynamical system too predictable or too slow to respond, yet the other extreme of too little organisation can pose challenges to the normal functions of a cell.

In microscale systems, ensembles of active biomolecular components (motors, filaments, muscles, sarcomeres, etc.) have to negotiate complex viscoelastic environments and time-dependent stresses. When system parameters evolve dynamically in time, this can often lead to bifurcations from steady-state dynamics to oscillatory behaviour (Tamayo et al. 2022; Kruse and Jülicher 2005; Westendorf et al. 2013). This can happen even in a minimal system comprising a single motor applying a follower force (i.e., applied tangentially) at the tip of an elastic filament that is pinned at one end. As the applied force increases, a Hopf bifurcation results, leading to an oscillatory instability (De Canio et al. 2017). This kind of ‘flutter’ instability was first described in macroscopic, high-Reynolds-number systems (think garden hose, or flapping flags) (Xie et al. 2016; Gregory and Paidoussis 1966; Argentina and Mahadevan 2005). The microscale version has since been proposed recently as a possible molecular mechanism for the onset of oscillations in beating cilia and flagella (Ling et al. 2018; Woodhams et al. 2022). The control of ciliary beating remains a very active topic of study and a major open question in the field, which we do not discuss at length here. Several other mechanisms have been studied extensively in the literature, with varying levels of experimental support (Lindemann and Lesich 2021; Brokaw 2009; Sartori et al. 2016).

While oscillatory dynamics can emerge naturally in this way, living systems likely evolved to harness control of this phenomenon for certain functions. A prominent demonstration of this is the sensing of sound in the vertebrate hearing organ, by bundles of mechanically activated stereocilia in the inner ear. In-depth analyses have suggested that self-oscillations amplify hearing responses close to the bifurcation threshold for instability (Martin et al. 2001). This is an active, nonlinear process that requires a constant flow of energy into the system to sustain the oscillations. Frequency tuning of self-oscillations further modifies the properties of the hair bundles, rendering them selectively sensitive to certain signal-frequency ranges. The exact mechanism and site of sensory amplification in hair cells is debated (Mhatre 2015), and it remains to be elucidated whether oscillations are an artefact or mechanism of active sensing in different systems, including the insect chordotonal organ (Göpfert and Hennig 2016).

The existence of oscillations also permits the possibility of their entrainment by other oscillators, cycles, or signalling events, notably in neural dynamics. The firing of an individual neuron is itself poised at criticality, as rationalised in seminal work by Hodgkin and Huxley (1952). The dynamics can be captured by a system of differential equations involving the time rate of change of the membrane potential. The system is excitable; intuitively, this means that even small (but above threshold) fluctuations in the balance of ionic fluxes can result in large deviations of the membrane potential or spiking event, where membrane depolarisation is followed by repolarisation. For certain parameter combinations, firing can occur at regular intervals, and the single neuron can thus be considered as an excitable oscillator (Stiefel and Ermentrout 2016; Rinzel and Ermentrout 1998). In larger animals, networks, circuits, or systems of neurons underpin diverse and complex sensorimotor rhythms, such as walking, running, or hunting. The rich dynamics of neural synchronization between different brain regions, or even avalanche events, are critical to information propagation and the proper organisation of brain function. The activity of oscillators can therefore enable entrainment on a global or collective scale, and in certain cases, as we have seen in the previous section, amplify sensory functions.

## Navigation strategies of microswimmers

Next, we discuss the significance of active oscillations, which characterise the self-propulsion strategies of most microscopic organisms, in the context of two distinct navigation algorithms, one stochastic and the other deterministic.

- Stochastic search in a colonial choanoflagellate

Due to their small physical size, most prokaryotes (with some exceptions) are severely constrained by thermal rotational diffusion and are therefore unable to sustain any given migration or swimming path for a sufficiently long time for effective signal integration (Berg 1984; Wan and Jékely 2021). Consequently, such microbes can only access stochastic search strategies, such as the classic run-and-tumble chemotaxis of *E. coli*. The latter is not generally a true *taxis* as the direction of swimming does not follow the chemical gradient; instead, the cell suppresses the tendency to tumble (associated with path reorientation) when migrating up the gradient towards a chemoattractant (Berg and Purcell 1977). Over long times, this biases the movement of the organism without the need for steering. Another common strategy is to bias the reorientation angle after tumbling, instead of the run speed, depending on the direction of the gradient (Saragosti et al. 2011). Detailed modelling and inference methods can be used to estimate the rotational diffusion associated with the active motility strategies of such micron-sized cells (Seyrich et al. 2018).

A recent biophysical study revealed that the choanoflagellate *Salpingoeca rosetta* adopts a stochastic strategy for aerotaxis—the biased migration towards oxygen (Kirkegaard et al. 2016a). This is somewhat surprising, since colonial eukaryotes like *S. rosetta* are certainly large enough to steer effectively (Wan and Jékely 2021), so it is unclear why in the transition to multicellularity, choanoflagellates have not evolved to steer. The answer may well lie in the stochastic morphology of *S. rosetta*: a facultative multicellular organism, where the number of cells per colony is highly variable, encompassing unicellular as well as multicellular forms, and the ability to switch between forms. A stochastic search may be a consequence of both the highly stochastic beating of the individual flagella, and the inability of the flagella to coordinate their activity over the entire colony, with no obvious relationship between colony size and fidelity of stochastic navigation (Kirkegaard et al. 2016b). In contrast, green algae of similar size do coordinate their flagella for processes such as phototaxis, as we shall discuss in the next case study. Genomic evidence places choanoflagellates as an important sister group to the metazoa (King 2004; Ros-Rocher and Brunet 2023). It is of interest that these organisms appear to have retained the more ancestral stochastic navigation strategy rather than evolve additional mechanisms for intercellular flagellar coordination or communication.

Another important feature is the demonstration of *logarithmic sensing* in choanoflagellate aerotaxis. Using a combination of fast gas-exchange experiments and simulations, Kirkegaard et al. (2016a) showed that *S. rosetta* effectively discriminates oxygen gradients in low-concentration environments by logarithmic sensing. In a coarse-grained, Keller–Segel-type description of the population dynamics, the drift velocity can be written as $V = v_\text {drift}\tanh \left( \frac{\gamma }{C}\frac{\textrm{d}C}{\textrm{d}y}\right) \textbf{e}_y$, for a perfectly vertical gradient in the $\textbf{e}_y$-direction, where $\gamma$ is a constant and $\tanh$ is the hyperbolic tangent function. Here, the speed is tuned not to the absolute gradient of the signal $\nabla C$ but instead to the change in signal relative to the background $\nabla C/|C|$ (in other words, to $\nabla \log C)$. (Here, $\nabla$ denotes the Del operator, so that $\nabla C = \textrm{d}C/\textrm{d}y$ in 1D, or $\nabla C = (\partial C/\partial x, \partial C/\partial y, \partial C/\partial z)$ in 3D.) In this interpretation of the experimental data, the individual swimming colony modulates its swimming direction stochastically in response to the gradient, rather than its swimming speed *v*. At long times, the propulsion strategy leads to $v_\text {drift}=v\left\langle \sin \theta \right\rangle$, where $\theta$ denotes the orientation of the individual colony and $\left\langle \cdot \right\rangle$ denotes the ensemble average. The distribution of turning angles in time $\Delta t$: $\Delta \phi = |\phi (t+\Delta t)-\pi /2|-|\phi (t)-\pi /2|$ was measured experimentally and confirmed to be centred at $\Delta \phi =0$ (Kirkegaard et al. 2016a). This produces a noisy search pattern with as many wrong turns as right turns (Fig. 2d).

This type of nonlinear sensing is found in many sensory systems operating both at the low- and high-signal limit (fold-change detection); this is analogous to why the sensitivity of the human ear to sounds is also quantified on a logarithmic scale (in decibels). From the perspective of saturating sensors at the microscale (Endres and Wingreen 2008), the error in the measured concentration gradient scales with the absolute concentration itself, so the limit of detection must decrease with absolute concentration. This is also known as *Weber’s law of sensory perception* (Stevens 1957). The physiological need to operate at the ‘sensory limit’ can significantly constrain the design of the signalling system that underlies these sensory functions, particularly at cellular scale, where thermal fluctuations are significant. Although the biochemical transduction pathway responsible for choanoflagellate aerotaxis has not yet been elucidated at a molecular level, it is possible that it shares some features with *E. coli* chemotaxis, where robustness is suggested to emerge from network design, and rather insensitive to exact system parameters (Barkai and Leibler 1997).

b. Deterministic steering in algae and sperm

In the above, the individual *S. rosetta* colony’s noisy helical trajectory (on the timescale of stochastic flagellar oscillations) was not accounted for, but yet could still play a role in the eventual stochastic search strategy. For over 100 years, helical navigation has been conjectured to be important for navigation in motile microeukaryotes (Jennings 1902), where the interplay between the organism’s intrinsic helical swimming path and the resulting oscillatory sampling of a directional signal leads to deterministic steering towards the stimulus, this time a true taxis (Crenshaw 1993). Recent experimental and theoretical studies have demonstrated deterministic steering in sperm chemotaxis (Kashikar et al. 2012; Jikeli et al. 2015; Kaupp and Alvarez 2016) as well as in the phototaxis strategies of diverse green algae (Cortese and Wan 2021; Drescher et al. 2010). Steering is possible despite active fluctuations occurring at the scale of individual flagella, which can surpass thermal fluctuations (Ma et al. 2014; Wan and Goldstein 2014).

Photosynthetic green algae, such as *Chlamydomonas reinhardtii* and *Volvox carteri*, have optimised their sensorimotor system for steering towards light (Yoshimura and Kamiya 2001). Unlike *S. rosetta*, both species coordinate their flagella (either in a pair or in a collective) to produce smooth helical swimming. Using a variety of experimental approaches to deliver pulsed light stimuli, the strongest frequency response in the motor system of these organisms was shown to be elicited at the self-rotation frequency (of helical swimming) (Leptos et al. 2023; Drescher et al. 2010; Schaller et al. 1997; Hegemann 1997). Moreover, in the biflagellate *C. reinhardtii*, the response delay time from signal perception to modulation of flagella activity also coincides with the timescale required for the cell’s mean flagellar beat plane, which lags behind the eyespot location, to rotate in line with the stimulus direction (Fig. 2). Another alga, *Euglena gracilis*, exhibits an adaptive phototaxis strategy and follows strikingly polygonal trajectories (varying in order from 3 to 5) in response to sudden increase in light intensity (Tsang et al. 2018). In all cases, the relationship between the sensor and detector can be encoded by $I(t) = I_0(\textbf{I}\cdot {\hat{\textbf{s}}})H(\textbf{I} \cdot \hat{\textbf{s}})$, where $\textbf{I}$ is the stimulus direction in the lab-frame, $(\textbf{I}\cdot {\hat{\textbf{s}}})$ is the stimulus projected onto the direction of maximal sensitivity of the detector (e.g., eyespot direction), and *H* is a Heaviside step function, which is equal to 0 when the argument is negative and 1 otherwise. The step function accounts for periodic shading of the detector as the detected intensity is zero until the eyespot has rotated to face the stimulus. When the organism oscillates along helical paths, the perceived stimulus also oscillates. Depending on the nature of the motor apparatus, *I*(*t*) then modifies the swimming behaviour accordingly.

To fulfil other forms of taxes, a dedicated directional sensor may not be required, as in the case of chemotaxis of animal sperms. Although spatial heterogeneities in the distribution of chemoreceptors and ion channels cannot be ruled out. In the extensively studied system of the sea urchin *Arbacia punctulata*, freely swimming sperm were placed in chemical landscapes and their 3D behavioural responses to caged resact, a potent chemoattractant for sea urchin sperm, released by photolysis were tracked by digital holography (Jikeli et al. 2015). In a simplified model of these measurements, the basic 3D flagellar waveform can be characterised by a constant twist and curvature $\kappa (s,t) = K_0+A\cos (\omega t-\lambda s)$ at time *t* and arclength *s*, where *A* is the wave amplitude, $\omega /\lambda$ the wave speed, and $K_0$ the mean curvature. Such a beat produces realistic superhelical paths (as well as twisted ribbons). Due to the periodic trajectory, a spatial concentration profile of chemoattractant $C(\textbf{x},t)$ is converted to a temporal stimulus at the location $\textbf{x}(t)$ of the sperm via $S(t) = C(\textbf{x}(t))$; this intracellular signal then induces a change in the path curvature $\kappa (t)$, with an adaptive time-delay (Friedrich and Jülicher 2007; Kashikar et al. 2012). For step-on responses (swimming up the chemical gradient), it has been shown both theoretically and experimentally that a small periodic modulation of the flagellum curvature leads to the smooth alignment of the helix axis $\textbf{h}$ with the gradient in a deterministic rather than stochastic manner. That is, the change in direction of the helix axis $d\textbf{h}/dt$ is biased towards the direction of $\nabla _\perp C = \nabla C-(\nabla C\cdot \textbf{h})\textbf{h}$, the component of the chemical gradient that is perpendicular to the initial helix orientation. The turning response is also Ca$^{2+}$-dependent, but not solely on the intracellular calcium concentration (Wood et al. 2007). Finally, we note that periodic dynamics are also involved in 2D exploration, for example with sperm swimming near boundaries or interfaces, often along circular trajectories instead of helices.

Despite the intrinsic differences between exploration of chemical versus photonic cues (binding of disperse molecules versus interception of photons), both sperm chemotaxis and algal phototaxis are examples of *helical klinotaxis* (Friedrich and Jülicher 2007; Crenshaw 1993). In both cases, the axis of the swimming helix gradually reorients until it is aligned with that of the stimulus gradient (Fig. 2d): while the baseline gradient controls the global response magnitude, the fast oscillations occur at the frequency of flagellar beating. By combining sensory adaptation (high-pass) with temporal integration (low-pass), klinotactic steering operates as a bandpass filter (Yoshimura and Kamiya 2001) This can result in reafferent sensorimotor coupling, as introduced in the previous section, where a feedback between the self-generated oscillatory signal and the stimulus produces a deterministic steering response with in-built error correction akin to a servomechanism.

The adaptive coupling between a microswimmer’s angular rotation and the vectorial stimulus is important for navigation. This simple mechanism is exemplified by its action in single microswimmers, but also exists in the neural circuits of animals (Jékely et al. 2008; Fraenkel and Gunn 1941; McHenry and Strother 2003; Kane et al. 2013). The self-generated gradients produced by an organism’s self-movement may also form the basis of other, lesser-explored navigation behaviours, such as rheotaxis and gravitaxis of planktonic marine larvae (Wheeler et al. 2019; Takeda-Sakazume et al. 2022; Jékely et al. 2021). Intriguingly, directional navigation can even emerge spontaneously in the case of oscillatory rheotaxis (upswimming against an external flow) in active artificial microswimmers (Dey et al. 2022), where oscillations are controlled by the interaction between the boundaries and the self-generated gradients in surfactant produced by the otherwise spherical, isotropic droplets. For a navigating animal, its undulatory movements can produce lateral oscillations that enable temporal sampling, and the ability to distinguish between step-up and step-down responses to stimuli. In movement ecology, achieving a fine balance between exploration (side-to-side movement) versus exploitation (direct migration from A to B) could be integral to the stochastic search patterns of migrating animals and even humans (Clement et al. 2023; Bartumeus and Levin 2008; Volchenkov et al. 2013), often with signatures of multiple oscillation frequencies in the dynamics.

## Discussion

### Coordinating oscillations for motility

We have demonstrated that at the cellular scale relevant for microscale navigation, oscillations and oscillatory dynamics emerge naturally, and are a powerful means to both encode and transduce information in space and time. They enable small cells and organisms to embody a mechanical intelligence about their environment whilst soliciting minimal involvement from the ‘brain’ or central-processor equivalent. By embedding periodic signatures in patterns of locomotion and sensing, they allow for coordinated or synchronized actions between different parts of the same organism, or between different organisms. While the ubiquity of oscillatory processes is undeniable, care should be taken not to ascribe function in cases where there may be none. Careful theoretical analyses and experimental studies are needed to determine whether true function exists in any given context.

In the course of evolution, active oscillatory dynamics in cells may have arisen accidentally but subsequently became preferentially selected. For example, motile cilia beating may have emerged when the first molecular motors that were originally intended for surface or intraflagellar transport (Bloodgood 2010) spontaneously coordinated within an elastic axoneme to produce self-sustained oscillations (Jülicher and Prost 1997). Over time, cells then repurposed these cilia oscillations and diverse stroke patterns exclusively for motility generation, due to their vastly superior function when navigating through fluids and other complex environments. Here, swimming relies on the cyclic deformation of parts of the organism’s body (e.g., the appendages), as indeed do the locomotion strategies of most terrestrial and aquatic animal species (Dickinson et al. 2000). The ubiquity of helical swimming in microbial navigation can be explained by noting that for periodic deformations in the body frame of the swimmer in a low-Reynolds number environment, superhelical swimming trajectories are the most general solution that satisfies the correct equations of fluid dynamics, since any asymmetric stroke will result in a non-zero translation and rotation after each stroke cycle (Rossi et al. 2017). This naturally leads to two distinct timescales encoded in the organism’s movement patterns, a fast timescale on the order of the self-deformations (e.g., cilia beat frequency), and a usually slower timescale over which the ’slow’ helical turns are completed (Fig. 2). For *Chlamydomonas*, typical rotation frequencies are 1–2 Hz, while flagellar beat frequencies are 50–70 Hz. As we have highlighted in the previous section, helical swimming has further downstream benefits for stimulus-oriented navigation. Helical klinotaxis obeys a similar fundamental physical principle, whether for organisms with largely symmetric body shape (Cortese and Wan 2021; Leptos et al. 2023; Jékely et al. 2008), or those with obvious body asymmetries (Rossi et al. 2017; Jikeli et al. 2015). In the case of helical chemotaxis, successful gradient following occurs for a wide range of system parameters (Lange and Friedrich 2021), again demonstrating underlying system robustness. Thus, this mechanism of converting spatial to (self-generated) temporal gradients is highly robust to noise, and forms the basis of a versatile and evolutionarily ancestral search strategy that is agnostic to the precise morphology of the organism.

Wherever multiple appendages are involved, how these are coordinated and controlled in different organisms remains an intriguing open question (Wan 2018; Gilpin et al. 2020). Not only are the control strategies system-dependent, they are also dynamic and sensitive to environmental changes (Quaranta et al. 2015; Klindt and Friedrich 2016). In many-cilia systems, hydrodynamic interactions can dominate the interactions between neighbouring appendages (Brumley et al. 2014; Vilfan and Jülicher 2006; Elgeti and Gompper 2013), whereas in systems with a small number of precisely positioned cilia, active intracellular mechanisms are important (Guo et al. 2021; Wan and Goldstein 2016). Continuing to draw parallels between microbial and animal navigation, we can identify deep similarities between microbial motility, in which modular cellular components are coupled dynamically with capacity for error correction, and motor programming in animal movement (Grillner et al. 1991; Collins and Richmond 1994). The limbs or segments of a locomoting animal’s body often maintain deterministic phase relationships; surprisingly, this is also observed in the gaits of algal flagellates (Wan and Goldstein 2016), and to a lesser extent in the stochastic gaits of walking ciliates that use cirri (bundled cilia) to move on surfaces (Larson et al. 2022) This raises the possibility that the ancestral analogue of central pattern generators (CPGs)—ubiquitous in animal locomotion Marder and Bucher (2001)—may have arisen in the first instance in the single-celled eukaryote, purely from the necessity of coordinating multiple oscillators for motility and navigation (Wan 2020). These basic mechanisms are then elaborated in the complex nervous system architectures of animals, where intrinsically oscillatory appendage actuation obeys different network topologies, and regulation by servomotors (Cheng 2022, 2023). CPG-inspired circuits also underlie the many designs for robots modelled on the locomotor patterns of animals Ijspeert (2008).

**Fig. 3:**
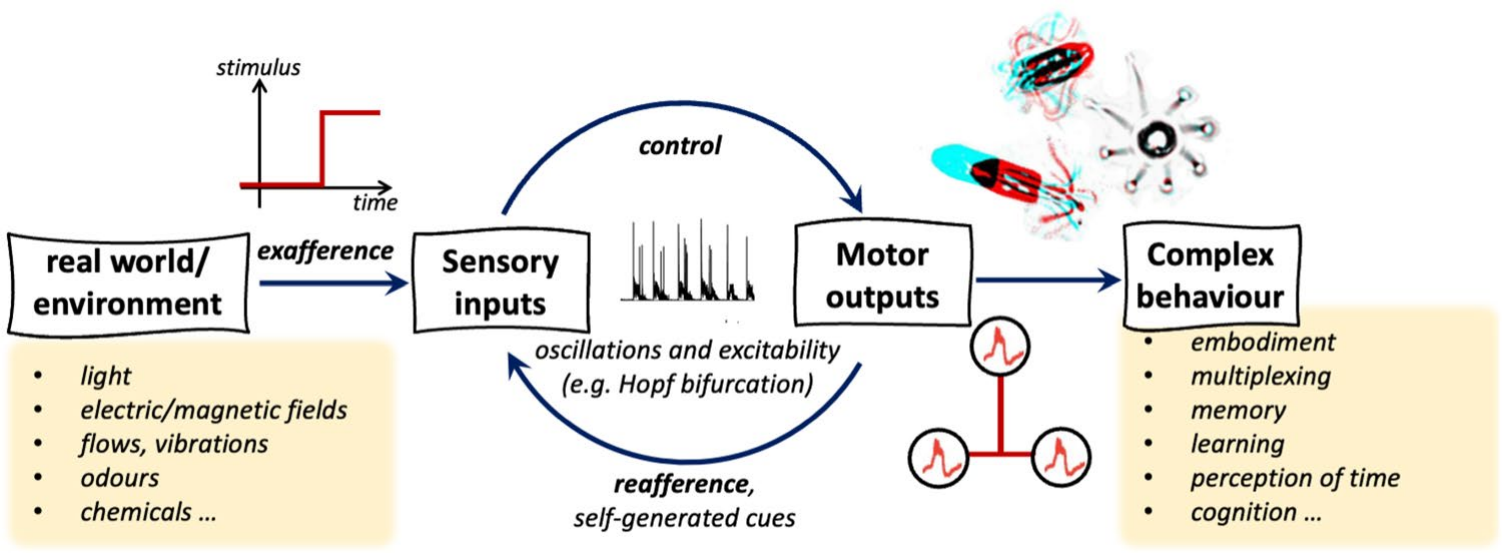
Animal cognition may have first evolved as a means to coordinate self-movement. Since oscillatory (in contrast to static inputs) have high-information content, microscopic organisms may have harnessed oscillatory dynamics and self-excitability to generate novel cognitive functions

### Motility, cognition, and perception of the self

We conclude our discourse by returning to the very notion that motility may have predated cognition itself (Fig. 3). As we seek to understand and trace the deep evolutionary origins of animal cognition, there is growing consensus that neural systems first evolved to coordinate self-movement, while sensory modulation arose only later (Goodrich 2010). Central to biological cognition is an organism’s perception of time, which may be embodied in the multiplexed oscillations of the body, effectively turning the body into a clock. At the macroscale, this has been demonstrated in rodents that were given a reward if approached after a fixed interval: instead of remaining at rest between events, the animals developed stereotyped motor routines that improved the accuracy of time-keeping (Safaie et al. 2020). The notion that ‘*each oscillatory cycle is a temporal processing window*’ (Buzsáki 2006) does not have to apply exclusively to neural systems; in contrast to static or constant-amplitude signals, a wave or a fluctuating signal can be intrinsically directional. I suggest that these features enable even microscopic organisms to perceive the arrow of time.

By exploiting the intrinsic properties of active systems close to a critical point, living systems can access both stable dynamics yet also rapid responses to external cues, as we saw in the oscillatory instabilities of beating cilia. Further, oscillatory inputs may have high information content, with the ability to buffer as well as filter signals. These conceptual traits are paralleled in excitable oscillators and neurons (Stiefel and Ermentrout 2016), as they are in the dynamics of the motile single cell. For decades, the ciliate *Paramecium* has been referred to as ‘a swimming neuron’ (Kung and Saimi 1985), with renewed interest in exploring the extent of this interpretation in recent years (Brette 2021; Elices et al. 2023). By generating and controlling repetitive spikes, or routines in activity, single cells can keep time, generate, and store memories, and even learn (Fig. 3). This type of learning typically manifests at timescales (short) relevant for cellular physiology, in contrast to inherited traits or generational adaptation which occurs over evolutionary timescales (long). These hidden intracellular signatures often manifest as changes in the observed motile behaviours of these organisms. Examples include associative learning in the avoidance behaviour of *Stentor* (Rajan et al. 2023; Gershman et al. 2021) and galvanotaxis in three species of free-living amoebae (Carrasco-Pujante et al. 2021), the stochastic hunting strategy of the protist *Lacrymaria*, in which space is repetitively and densely sampled (Coyle et al. 2019), and the rhythmic dynamics of ciliate escape from dead ends (Kunita et al. 2014), though the biochemical origins of the latter is disputed (Ishikawa and Kikuchi 2018).

Excitable membrane dynamics and other threshold phenomena may have contributed significantly to the origin of eukaryotes, which co-evolved with diversification of cilia function and increased complexity in the cognitive capabilities of cells. Thus, with motility, so came cognition; we should be left in no doubt that the microscopic world holds unique and invaluable insights into how sensory and cognitive processes are encoded in living systems.

## Data Availability

Not applicable.
